# Beyond the Cut: Understanding Non-Suicidal Self-Injurious Behavior Through Trauma and Emotional Dysregulation

**DOI:** 10.1192/j.eurpsy.2026.11279

**Published:** 2026-07-31

**Authors:** D. R. Gupta, S. Prasad

**Affiliations:** 1 Psychiatry, NYC Health + Hospitals/Metropolitan Hospital Center; 2 Psychiatry, BronxCare Health System, New York, United States

## Abstract

**Introduction:**

Non-Suicidal Self Injurious Behavior (NSSIB) is defined as the deliberate infliction of harm to one’s own body without suicidal intent, often to manage overwhelming psychological distress, regulate emotions, or address interpersonal conflicts. It frequently appears among hospitalized patients, positioning psychiatrists at the forefront of screening and management.

**Objectives:**

This submission presents a unique case of non-suicidal self-injurious behavior (NSSIB) driven by the substitution of substance-induced euphoria and reviews current literature on the motivations underlying NSSIB, as well as approaches to screening, risk stratification, and management.

**Methods:**

A case-based descriptive analysis was conducted. The case involved a 19-year-old woman with autism spectrum disorder, major depressive disorder, and substance use disorders (cannabis, heroin, ketamine, and tobacco) who presented with extensive superficial self-inflicted cuts on her forearms, legs, and thighs. She attributed her self-injurious behavior to traumatic childhood experiences, explaining that the sight of blood alleviated her anxiety and became her primary coping mechanism in maintaining sobriety. The literature review examined motivations for NSSIB, screening protocols, risk stratification, and management strategies.

**Results:**

Psychiatrists are uniquely positioned in emergency and inpatient settings to evaluate NSSIB early, conduct comprehensive risk assessments—including evaluating suicidal risk—and explore patient-specific motivations. The DSM-5-TR classifies NSSIB under “Other Conditions That May Be a Focus of Clinical Attention,” emphasizing its clinical importance independent of other psychiatric diagnoses. Known risk factors include adverse childhood experiences, bullying, abuse/neglect, and comorbid psychiatric conditions such as mood, personality, or eating disorders (Plener et al., 2018). NSSIB can also signify an elevated risk for future suicide (Cipriano et al., 2017). Effective management requires a nonjudgmental approach, psychotherapeutic interventions, trauma-informed care, and, where appropriate, pharmacological treatment targeting underlying psychiatric disorders (Ougrin et al., 2015). Structured screening processes are vital for timely identification of at-risk individuals and prompt initiation of appropriate interventions.

**Conclusions:**

Psychiatrists often serve as the first point of clinical contact for individuals presenting with NSSIB. This case underscores the importance of a tailored, trauma-informed treatment plan that addresses both the emotional and neurobiological dimensions of NSSIB. The accompanying literature review highlights the need for further research to optimize screening protocols, promote healthier coping strategies, and support multidisciplinary collaboration in managing NSSIB.

**Disclosure of Interest:**

None Declared

